# Jump Control Based on Nonlinear Wheel-Spring-Loaded Inverted Pendulum Model: Validation of a Wheeled-Bipedal Robot with Single-Degree-of-Freedom Legs

**DOI:** 10.3390/biomimetics10040246

**Published:** 2025-04-17

**Authors:** Jingsong Gao, Hongzhe Jin, Liang Gao, Yanhe Zhu, Jie Zhao, Hegao Cai

**Affiliations:** School of Mechatronics Engineering, Harbin Institute of Technology, Harbin 150080, China; 19B908036@stu.hit.edu.cn (J.G.); yhzhu@hit.edu.cn (Y.Z.); jzhao@hit.edu.cn (J.Z.); hgcai@hope.hit.edu.cn (H.C.)

**Keywords:** jump control, nonlinear spring, wheeled-bipedal robot, trajectory planning

## Abstract

Jumping is a fundamental capability for wheeled-bipedal robots (WBRs) navigating unstructured terrains, with jump height and stability serving as indicators of the robot’s environmental adaptability. However, existing trajectory planning methods demand high output capacity from the joints and fail to balance computational load with trajectory tracking performance. This limitation hinders most robots from experimental validation. To address these challenges, this study presents an optimized virtual model, trajectory planning strategy, and control method. These solutions enhance both the height and stability of jumps while ensuring real-time execution on physical robots. Firstly, inspired by the human jumping mechanism, a Nonlinear Wheel-Spring-Loaded Inverted Pendulum (NW-SLIP) model was originally proposed as the virtual model for trajectory planning. The jump height is increased by 3.4 times compared to the linear spring model. Then, cost functions are established based on this virtual model, and the trajectory for each stage is iteratively optimized using Quadratic Programming (QP) and a bisection method. This leads to a 21.5% increase in the maximum jump height while reducing the peak joint torque by 14% at the same height. This significantly eases execution and enhances the robot’s trajectory-tracking ability. Subsequently, a leg statics model is introduced alongside the kinematics model to map the relationship between the virtual model and joint space. This approach improves trajectory tracking performance while circumventing the intricate calculation of the dynamics model, thereby enhancing jump consistency and stability. Finally, the proposed trajectory planning and jump control method is validated through both simulations and real-world experiments, demonstrating its feasibility and effectiveness in practical robotic applications.

## 1. Introduction

Wheeled-bipedal robots (WBRs) combine the high speed and efficiency of wheels with the adaptability of legs [1,2], enabling flexible navigation on the ground [3,4,5,6]. When faced with unstructured terrains, jumping has emerged as one of the essential functions for advanced WBRs to overcome obstacles, such as Ascento [7,8] and Ollie [9,10]. However, research on jump control for WBRs is relatively limited. Insights can be drawn from single-legged robots with the decoupling of legs and wheels. Researchers [11,12] simplified these robots into a centroid model, with its trajectory planned through kinematics and dynamics analysis. Nevertheless, developing an accurate dynamics model for a complex robot platform remains challenging. Other researchers established simplified virtual models to characterize the robot’s fundamental dynamics. The trajectory is planned based on these virtual models and mapped to the joint space. For example, Luo [13] and Piovan [14] utilized the SLIP model to plan the trajectory, achieving stable jumps. Mathis [15] further proposed a two-mass spring model that improved accuracy by considering the lower centroid during jumps. Additionally, some studies have formulated jumping trajectories based on optimal control [16] and stability [17].

In the realm of WBR jump control, Chen [18] proposed a wheeled-spring-loaded inverted pendulum model to characterize the dynamics of the robot during jumping, with trajectory planning for the stance and flight phase handled through a quadratic program. Zhuang [19] introduced a wheeled-bipedal jumping dynamical model to optimize height control and the Bayesian optimization for torque planning. This resulted in an 82.3% reduction in height error and a 26.9% decrease in energy expenditure. Hao [20] also simplified the robot into two masses, with the run-and-jump trajectory plotted using nonlinear programming. Li [21,22,23,24] divided the jumping sequence into four stages and conducted trajectory planning for each stage based on the Two Mass Spring Damp Inverted Pendulum model, examining it on the WLR-3P. Except for Li’s work, which was implemented on a robot using a more energy-dense hydraulic drive, the other three studies were limited to simulations. These models referenced above simplify the WBR into upper and lower mass blocks connected via a linear spring. For clarity, they are collectively referred to as the Linear Wheel-Spring-Loaded Inverted Pendulum (LW-SLIP) model in this paper. Due to the difficulty in dynamics modeling and the non-negligible mass of the wheel, the LW-SLIP model is employed as the virtual model to plan the jumping trajectory of WBRs. However, the model’s high demand for joint output capacity hinders most robots from experimental validation.

WBRs with single-degree-of-freedom legs (SDoF WBRs) offer simple control and lightweight design, though research on their jump control remains limited. The SDoF leg poses challenges due to the increased demand for joint output and the uncontrollable forward displacement of the fuselage centroid, which complicates stable jump execution. However, these characteristics render them suitable for validating the proposed jump control method.

The contributions of this study are summarized as follows. Firstly, a novel human-emulated Nonlinear Wheel-Spring-Loaded Inverted Pendulum (NW-SLIP) model is introduced [25,26,27]. The dual-mass model effectively captures the key dynamic characteristics of WBR, and the spring’s nonlinear characteristics result in a 3.4-fold increase in jump height. Then, cost functions are constructed based on the NW-SLIP model, with significant constraint conditions defined. The trajectory of each stage is iteratively optimized using Quadratic Programming (QP) and the bisection method, resulting in a 21.5% increase in maximum jump height. The peak joint torque at the same height is reduced by 14%, facilitating trajectory tracking. In addition to utilizing the kinematic model for trajectory tracking, a leg statics model is incorporated to calculate feedforward joint torques, improving trajectory tracking performance. Compared to the dynamics model, this approach offers superior computational efficiency and enhanced real-time processing capability. It also reduces the impact of sensor noise, ensuring greater consistency and robustness in jump performance. Finally, trajectory planning and jump control methods are validated on the designed WBR through both simulations and experiments. The results indicate that the NW-SLIP model is more compatible with the output characteristics of the jointed-leg structure, enabling higher jump heights. The proposed jump control method enables the robot to achieve high consistency and stability in jumping.

The rest of this paper is organized as follows: Section 2 describes the structural and hardware system of the designed WBR. Section 3 establishes the NW-SLIP model, kinematics model, and leg statics model. Section 4 introduces the trajectory planning and jump control method. Simulations and experiments are presented, respectively, in Section 5 and Section 6. Section 7 concludes this paper.

## 2. Structural and Hardware Design

**Figure 1:**
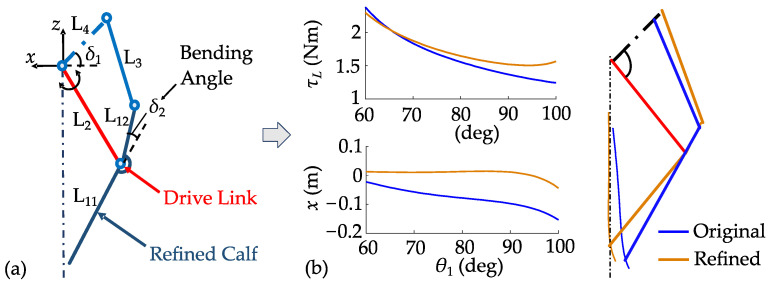
Optimization of leg configuration: (**a**) refined four-linkage; (**b**) optimization effect.

The forward displacement of the fuselage centroid due to leg extension induces pitch acceleration during jumping, which challenges the robot’s self-balance ability after landing. For the SDoF WBR, this displacement cannot be eliminated through control but can only be minimized through structural design, which is crucial for stable jumping. Consequently, the leg linkage configuration is optimized to minimize forward displacement at the foot end while ensuring adequate extension range and increasing minimum ground support force to improve jump height, as shown in Figure 1.

**Figure 2:**
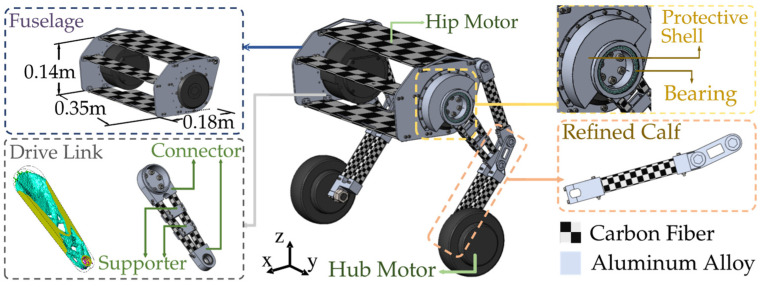
Design process of WBR.

The four-bar linkage is optimized by introducing a bending angle at the calf. The leg remains controlled by the hip joint through the drive link. The design variables include the lengths of the remaining links, the included angle between L_4_ and the fuselage’s horizontal axis, and the bending angle. Assuming the length of the drive link is 1 m and the ground support force is 1 N, the original linkage exhibits a forward displacement range of −0.15 m to −0.02 m, whereas the refined four-linkage displays a slight deviation, thus enhancing jumping stability. In addition, the linkage maintains a similar vertical extension range as before, with a slight increase in the minimum ground support force, which is beneficial for improving jump height, as shown in Figure 2.

**Figure 3:**
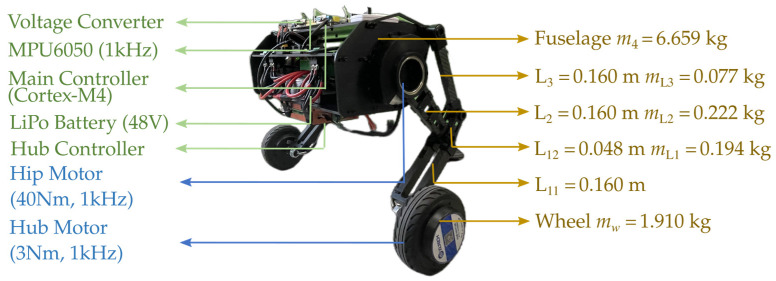
Prototype platform of the designed WBR.

The designed WBR is constructed from carbon fiber and aluminum alloy to reduce weight. Lightweight leg linkage minimizes the impact of leg movement on the overall centroid. The fuselage’s centroid is designed close to the hip joint to reduce the range of the target pitch angle, thus minimizing the pitch acceleration and enhancing stability during jumps. Leg linkages are installed outside the fuselage to increase the distance between two wheels, improving the stability margin in the frontal plane. The design of the drive link is based on topology optimization results, forming an integrated closed-loop structure using metal connectors on both sides and two carbon fiber rods. Two metal support components are incorporated to simulate the support points from the topology optimization results. The protective shell is designed to prevent the hip motor from being damaged by excessive overturning torque. It is fixed on each side of the fuselage and connected to the drive link through a bearing, as shown in Figure 3.

The main controller of the system uses a Cortex-M4 processor (Guangzhou Xingyi Electronics Technology Co., Ltd., Guangzhou, China), which provides strong computational capabilities to support efficient real-time control tasks. The drive system includes a high-power hip motor with a rated torque of 40 Nm (Jiangxi Xintuo Enterprise Co., Ltd., Nanchang, China) and a hub servo motor (Fujian Zhongyan Electric Motor Co., Ltd., Quanzhou, China) with a peak torque of 3 Nm. The hip motor communicates directly with the main controller, while the hub motor communicates via a motor driver. Each motor is equipped with sensors, such as encoders, to provide precise feedback on position, velocity, and torque, with a maximum communication frequency of 1 kHz. The system is powered by two series-connected LiPo batteries (Shenzhen Grepow Electronic Co., Ltd., Shenzhen, China), which supply power to the drive unit and main controller through a voltage conversion module. Additionally, the robot is equipped with an MPU6050 sensor (Guangzhou Xingyi Electronics Technology Co., Ltd., Guangzhou, China), which provides posture data at a sample frequency of 200Hz, delivering precise acceleration and orientation information. The overall control frequency of the prototype system is 280 Hz. The high control frequency and low sensor latency effectively reduce the impact of delays on jumping performance. The overall weight of the designed WBR is 11.47 kg. When the legs are fully retracted and the fuselage is at the lowest height, the hip motor angle is calibrated to 0 rad. The height of the fuselage ranges from 0.227 m to 0.425 m, with the hip angle ranging from 0 rad to 0.75 rad.

## 3. Jump Model Based on Nonlinear Spring

The NW-SLIP model is established for trajectory planning. The kinematics model and leg statics model are developed to define the mapping relationship.

### 3.1. NW-SLIP Model

**Figure 4:**
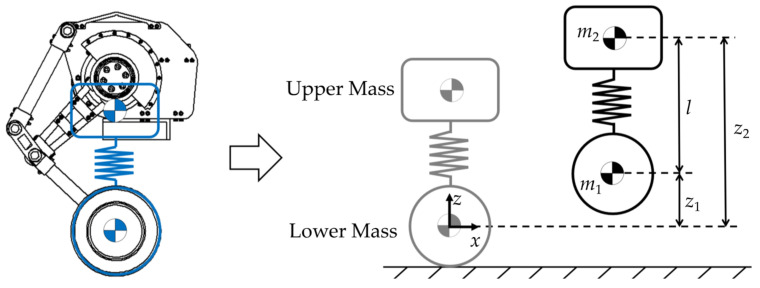
Nonlinear Wheel-Spring-Loaded Inverted Pendulum (NW-SLIP) model.

To accurately represent the key dynamic characteristics of the designed WBR, the NW-SLIP model is formulated as shown in Figure 4. The fuselage and leg linkage are considered the upper mass, while the two hub motors are considered the lower mass. These two masses are connected by a nonlinear spring, which deforms according to the movement of the hip joint. To facilitate trajectory planning, the forward-direction axis of the wheel’s centroid is considered as the reference line when it contacts the ground. Let *m*_1_ and *m*_2_ denote the centroids of the two masses. The corresponding heights of these centroids are denoted by *z*_1_ and *z*_2_, respectively. The distance between them is considered as the spring length, denoted by *l*.

To ensure the output monotonicity and computational efficiency, the expression of the nonlinear spring, including linear term and cubic term, is adopted as

(1) $$\left\{\begin{array}{l} H_{0}(k,l,l_{0})=m_{2}\ddot{l}-k(l_{0}-l)+m_{2}g=0\\ H_{1}(a,b,l,l_{0})=m_{2}\ddot{l}-a{\left(l_{0}-l\right)}^{3}-b(l_{0}-l)+m_{2}g=0 \end{array}\right.$$

where *l*_0_ represents the natural length of the spring, while *a*, *b*, and *k* denote the spring’s stiffness coefficients. For the sake of clarity and consistency in the subsequent discussion, *H*_1_(*a*, *b*, *l*, *l*_0_) is used to represent the constraints of the NW-SLIP model, whereas the constraints of the LW-SLIP model are referred to as *H*_0_(*k*, *l*, *l*_0_).

### 3.2. Kinematics Model of the Designed WBR

**Figure 5:**
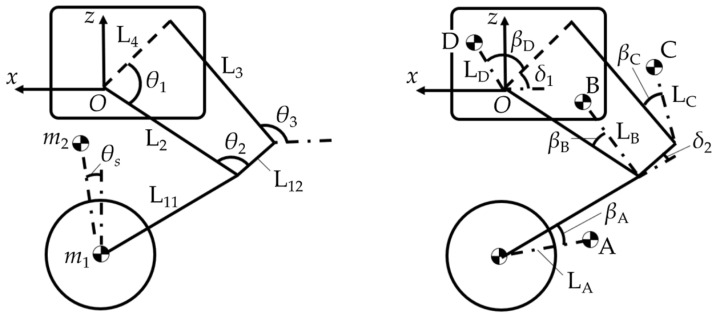
Kinematics model of the designed WBR.

The kinematics model is developed to calculate the target pitch angle and establish the mapping relationship between the length and velocity of the virtual spring, and the angle and angular velocity of the hip joint, as shown in Figure 5. Together, A, B, C, and D, which represent the centroids of each leg-link and the fuselage, respectively, constitute the upper mass.

The coordinates of the upper and lower masses are obtained as (*x*_1_, *z*_1_) and (*x*_2_, *z*_2_). Finally, the target pitch angle, length, and velocity of the virtual spring are determined:

(2) $$\left\{\begin{array}{l} \theta_{s}=\arctan(\frac{x_{1}-x_{2}}{z_{2}-z_{1}})\\ l(\theta_{1})=\sqrt{{\left(x_{2}-x_{1}\right)}^{2}+{\left(z_{2}-z_{1}\right)}^{2}}\\ \dot{l}(\theta_{1},{\dot{\theta }}_{1})=\frac{2[(x_{2}-x_{1})({\dot{x}}_{2}-{\dot{x}}_{1})+(z_{2}-z_{1})({\dot{z}}_{2}-{\dot{z}}_{1})]}{\sqrt{{\left(x_{2}-x_{1}\right)}^{2}+{\left(z_{2}-z_{1}\right)}^{2}}} \end{array}\right.$$

where

$$\left\{\begin{array}{l} x_{2}=\frac{2m_{A}x_{A}+2m_{B}x_{B}+2m_{C}x_{C}+m_{D}x_{D}}{2m_{A}+2m_{B}+2m_{C}+m_{D}}\\ z_{2}=\frac{2m_{A}z_{A}+2m_{B}z_{B}+2m_{C}z_{C}+m_{D}z_{D}}{2m_{A}+2m_{B}+2m_{C}+m_{D}} \end{array}\right.$$

### 3.3. Leg Statics Model

**Figure 6:**
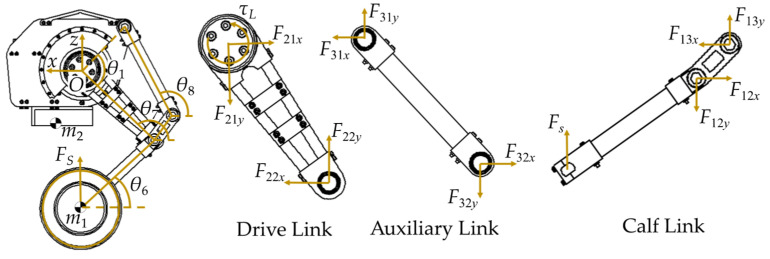
Leg statics model.

In this study, a feedforward torque is introduced to enhance trajectory tracking. Given the negligible rotational inertia of the lightweight leg links, a leg statics model is established to compute the mapping between spring force and joint torque, as shown in Figure 6. For high-frequency tasks, such as jump control, using a dynamics model would significantly increase computation time, adversely affecting the response speed and stability of the real-time control system. In contrast, the statics model provides sufficiently accurate joint torque predictions while greatly reducing computational complexity, making it more suitable for real-time applications. Additionally, the statics model is less susceptible to external disturbances and sensor noise, ensuring consistency and stability during jumps while also being cost-effective to model and offering greater scalability.

During self-balancing and uniform motion, the upper mass remains directly above the axle (i.e., *x*_1_ = *x*_2_), allowing *θ*_6_ to be obtained according to Equation (2). The leg statics model is established using the Newton–Euler method, and the force analysis is shown as follows:

(3) $$\left\{\begin{array}{l} F_{s}=F_{12y}-F_{13y}\\ F_{13y}=-F_{13x}\tan\theta_{8}=-F_{12x}\tan\theta_{8}\\ F_{s}L_{11}\cos\theta_{6}=L_{12}(F_{13x}\sin\theta_{7}-F_{13y}\cos\theta_{7})\\ \tau_{L}=F_{22y}L_{2}\sin\theta_{9}+F_{22x}L_{2}\cos\theta_{9} \end{array}\right.$$

where

$$\left\{\begin{array}{l} \theta_{7}=\theta_{6}+\delta_{2}\\ \theta_{8}=\pi +\theta_{7}-a\cos\frac{L_{12}^{2}+L_{3}^{2}-L_{2}^{2}-L_{4}^{2}+2L_{2}L_{4}\cos\theta_{1}}{2L_{12}L_{3}}\\ \theta_{9}=\theta_{7}+\theta_{2} \end{array}\right.$$

Finally, the relationship between the hip joint torque and spring force can be obtained as

(4) $$\tau_{L}=F_{s}L_{2}\left[L_{12}\sin(\theta_{7}-\theta_{8})\cos\theta_{9}+L_{11}\cos\theta_{6}\sin(\theta_{9}-\theta_{8})\right]/L_{12}\sin(\theta_{7}-\theta_{8})$$

**Remark** **1.** *As the fuselage’s height decreases, the ground support force generated by the jointed legs diminishes, while the force of the linear spring increases. This results in the underutilization of the joint’s output capacity in the LW-SLIP model. Research indicates that to achieve greater jump heights, humans maximize the ground support force during the thrust stage, which exhibits a nonlinear relationship with body height [25,26,27]. Consequently, the essence of the NW-SLIP model lies in simulating this efficient jumping mechanism to fully utilize joint output capabilities. From an algebraic perspective, it can also be demonstrated that a nonlinear spring can store more elastic potential energy under the same compression and force, thereby achieving a higher jump*.

(5) $$\left\{\begin{array}{l} △F_{e}=F_{e1}-F_{e2}=k\Delta l-a\Delta l^{3}-b\Delta l\\ △E_{e}=E_{e1}-E_{e2}=k\Delta l^{2}/2-a\Delta l^{4}/4-b\Delta l^{2}/2 \end{array}\right.$$

*where* ∆*l denotes the compression, F_e_*_1_ *and F_e_*_2_ *represent the spring forces, and E_e1_ and E_e2_ correspond to the elastic potential energies. When* ∆*F_e_* = 0*, the difference in elastic potential energy is given by*

(6) $$\Delta E_{e}=E_{e1}-E_{e2}=a\Delta l^{4}/4$$

*According to (6), when a is negative, the nonlinear spring stores more elastic potential energy, thus achieving a higher jump. Based on the nonlinear spring, the compression corresponding to the maximum force can be flexibly designed to adapt to the output characteristics of the joint, fully exerting the jumping capability of the robot.*

## 4. Trajectory Planning and Jump Control

**Figure 7:**
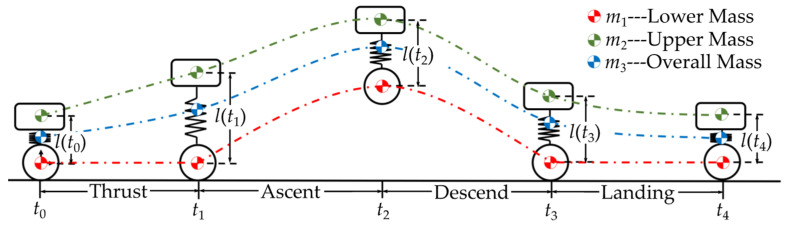
Four stages of the jumping sequence.

This section introduces the overall jump control method and the iterative optimization of trajectories for each stage. The jumping sequence is divided into four stages: thrust, ascent, descent, and landing (Figure 7).

During the thrust stage, the legs extend rapidly to provide kinetic energy for jumping. During the ascent stage, the overall centroid rises, and the obstacle-crossing height is further increased by retracting the legs. The descend stage stabilizes the legs and prepares for landing. The landing stage dissipates excess kinetic energy and adjusts the robot to a specified height. The transitions between stages are time-based, with switching instants defined as from *t*_0_ through *t*_4_. Taking the wheel center at *t*_0_ as the coordinate origin, the parameters of the designed WBR and virtual model are defined (Table 1).

The trajectory planning process takes the target jump height as input and aims to minimize joint torque. The spring lengths at the start and end times of each stage, along with the natural length and stiffness coefficients of the spring, are determined. From these, time-based curves for the trajectories of the centroids and spring force are derived. Thus, the trajectory planning of each stage can be regarded as a constrained nonlinear optimization problem.

### 4.1. Jump Control Method

**Figure 8:**
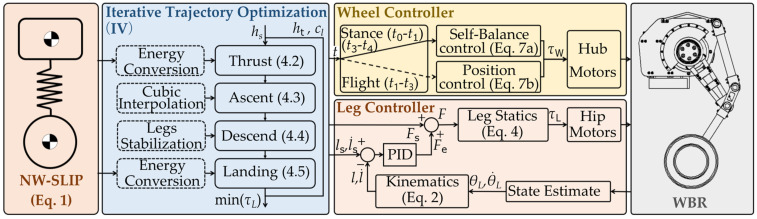
Schematic diagram of jump control method based on NW-SLIP model.

The overall control architecture is shown in Figure 8. The hip joint tracks the planned jump trajectory, while the wheel subsystem identifies its current phase based on time and subsequently calculates the required torque.

Initially, the jump trajectory is iteratively optimized based on the target height, yielding the planned spring length, velocity, and force. During the jump, while in motion, the takeoff speed and position are determined according to the planned trajectory. The legs reach the preset angle, and the robot begins to accelerate. Once the robot reaches the takeoff position under the preset speed, the jumping sequence is initiated. The leg controller determines the real-time spring length of the virtual model through state estimation and kinematics model. The required spring force *F_e_* for trajectory tracking is calculated using PID control, while the final spring force *F* is derived by combining the planned spring force *F_s_*. This final force is then mapped to the joint space utilizing the leg statics model. Incorporating feedforward spring force during spring length tracking can effectively enhance the robustness of jump control. It helps compensate for errors caused by variations in mass distribution, sensor noise, ground compliance, and other factors. The wheel controller manages state transitions according to the planned durations for each stage. When the wheel contacts the ground, a three-loop parallel PID control algorithm is applied, including an upright loop, a steering loop, and a position loop. When the wheel lifts off the ground, only the position loop PID control is used to stabilize the wheel:

(7) $$\tau_{w}=\left\{\begin{array}{l} \tau_{w1}=K_{p\theta }(\theta_{L})[\theta -\theta_{s}(\theta_{L})]+K_{d\theta }(\theta_{L})\dot{\theta }+K_{pp1}(\theta_{L})(x-x_{s})\\ \;\;\;\;\;\;\;\;+K_{dp1}(\theta_{L})(\dot{x}-{\dot{x}}_{s})\pm [K_{p\psi }(\psi -\psi_{s})+K_{d\psi }\dot{\psi }]\;\;\;\;\;\;\;\;\;\;\;\;\;\;\;\;\;\;\;\;\;\;\;\;\;\;,t\in [t_{0},t_{1}]\cup [t_{3},t_{4}]\\ \tau_{w2}=K_{pp2}(x-x_{s})+K_{dp2}(\dot{x}-{\dot{x}}_{s})\;\;\;\;\;\;\;\;\;\;\;\;\;\;\;\;\;\;\;\;\;\;\;\;\;\;\;\;\;\;\;\;\;\;\;\;\;\;\;\;\;\;\;\;\;\;\;\;\;\;\;\;,t\in (t_{1},t_{3}) \end{array}\right.$$

This prevents the wheel from overspeed rotation, which could affect the robot’s location and self-balancing after landing. The control parameters of the PID controllers are determined using the Z-N method [28,29].

### 4.2. Thrust Stage Trajectory Planning

In this study, the jumping trajectory is planned based on the NW-SLIP model using the cubic interpolation method, with cost functions formulated accordingly. To enhance clarity in the following discussion, we first provide a summary of the key variables used in trajectory planning along with their definitions, as shown in Table 2.

Leg retraction during flight can further improve the jump height and alleviate the strain on the hip motor. Thus, the target jump height *h_s_* (where *h* refers to the height of the wheel) is divided into target thrust height *h_t_* and target ascent height *h_a_*. The robot should reach *h_t_* at *t*_2_ without leg retraction or attain *h_s_* under the complete trajectory. Cost functions are formulated based on the NW-SLIP model to minimize the required joint torque while ensuring jump accuracy. To achieve the desired height of the robot’s overall centroid, the joints must generate the corresponding kinetic energy. Therefore, the support force coefficient *G*(*F_s_*, *l*_0_) is a more effective measure of the efficiency of the planned trajectory in utilizing joint output rather than the integral of torque. The trajectory planning for the thrust stage is cast as the following optimal control problem:

(8) $$\begin{array}{l} \min\;\;\;\;\;\;\Delta E_{s}^{2}(a,b,l,l_{0},t)+G^{2}(F_{s},l_{0})\\ s.t.\;\;\;\;\;\;\;H_{1}(a,b,l,l_{0})=0\\ \;\;\;\;\;\;\;\;\;\;\;\;\;\;q\in c_{l}Q,F_{s}\in N\\ \;\;\;\;\;\;\;\;\;\;\;\;\;\;F_{s}(t_{1})=-m_{1}g \end{array}$$

where

$$\left\{\begin{array}{l} E_{s}(a,b,l,l_{0},t)=\frac{1}{4}a{\left(l(t)-l_{0}\right)}^{4}+\frac{1}{2}b{\left(l(t)-l_{0}\right)}^{2}+m_{2}gl(t)+\frac{1}{2}m_{2}\dot{l}{\left(t\right)}^{2}\\ \Delta E_{s}(a,b,l,l_{0},t)=E_{s}(a,b,l,l_{0},t_{1})-E_{s}(a,b,l,l_{0},t_{0})\\ G(F_{s},l)=\frac{\max(F_{s})}{F_{\max}(l)} \end{array}\right.$$

The second cosntraint ensures that the trajectory adheres to the constraints of the NW-SLIP model. The coefficient *c_l_* further limits the maximum extension length during the thrust stage, creating space for leg extension in the ascent stage. The third constraint ensures that at *t*_1_, the lower mass is lifted by the spring force and is about to leave the ground. The stiffness coefficients and natural length of the spring, and the spring lengths at the start and end of the thrust stage are optimized as variables in the QP problem. After solving for these parameters, the trajectories of spring length, velocity, and force can be further determined.

### 4.3. Ascent Stage Trajectory Planning

The terminal state of the thrust stage determines the parabolic trajectory of the overall mass, and the duration of the ascent stage can be obtained. Since the NW-SLIP model lacks an analytical trajectory-time solution and the LW-SLIP model still underutilizes joint output, cubic interpolation is employed to plan trajectories based on the accelerations of the upper and lower masses.

(9) $$H_{2}(z_{1},c_{i},t)={\ddot{z}}_{1}(t)-c_{1}t^{3}-c_{2}t^{2}-c_{3}t-c_{4}=0$$

Then, the trajectory of the ascent stage can be determined through the following optimization process:

(10) $$\begin{array}{l} \min\;\;\;\;\;\;L^{2}(c_{i},t_{2})+G^{2}(F_{s},l)\\ \;\;s.t.\;\;\;\;\;H_{2}(z_{1},c_{i},t)=0\\ \;\;\;\;\;\;\;\;\;\;\;\;\;\;q\in Q,F_{s}\in N\\ \;\;\;\;\;\;\;\;\;\;\;\;\;\;{\dot{z}}_{1}(t_{2})=0 \end{array}$$

where

$$L(c_{i},t_{2})=\frac{1}{20}c_{1}t_{2}^{5}+\frac{1}{12}c_{2}t_{2}^{4}+\frac{1}{6}c_{3}t_{2}^{3}+\frac{1}{2}c_{4}t_{2}^{2}-h_{s}$$

The trajectory must satisfy the constraints outlined in (the first constraint without employing *c_l_* to impose restrictions on the state variables. When the overall centroid reaches its highest point, the lower mass reaches the target height, and the speed returns to zero.

### 4.4. Descend Stage Trajectory Planning

The descent stage is designed to prepare for landing. Its short duration increases the difficulty of leg extension. Trajectory tracking errors may cause premature ground contact, compromising jump accuracy and potentially leading to a significant impact. To mitigate this, the hips remain stationary during the descent stage, allowing more time for leg stabilization. The trajectory of the landing stage is planned based on the spring length at *t*_2_, which limits the magnitude of leg retraction in the ascent stage but is more conducive to a stable jump.

### 4.5. Landing Stage Trajectory Planning

The trajectory of the landing stage is also planned based on the NW-SLIP model. The optimization process is summarized as follows:

(11) $$\begin{array}{l} \min\;\;\;\;\;\;\Delta E_{s}^{2}(a,b,l,l_{0},t)+G^{2}(F_{s},l)\\ \;\;s.t.\;\;\;\;\;H_{1}(a,b,l,l_{0})=0\\ \;\;\;\;\;\;\;\;\;\;\;\;\;\;q_{1}\in Q_{1},F_{s}\in N \end{array}$$

Minimizing the energy error function ensures that all kinetic and gravitational energies are converted into elastic energy at the end of the landing stage, facilitating the robot’s transition into the self-balancing state. The jumping trajectory is obtained by optimizing the spring’s stiffness coefficients and natural length, and the spring lengths at the start and end of the landing stage. Taking *h_t_* and *c_l_* as variables, the trajectories of each stage are iteratively optimized. This process aims to minimize the hip motor’s maximum torque and speed, improving trajectory tracking feasibility. The bisection method is utilized to improve the iterative efficiency of the optimization process.

## 5. Jumping Simulation on the Designed WBR

The proposed trajectory planning and jump control method is validated in Adams (Mechanical Dynamics Inc., Los Angeles, CA, USA). Adams provides accurate modeling capabilities, optimizes jumping motion, and supports integration with other platforms, ensuring the feasibility and precision of control strategies and hardware designs [30,31]. The parameters of the designed SDoF WBR are set for simulation.

### 5.1. Simulation of Jumping Without Leg Retraction

**Figure 9:**
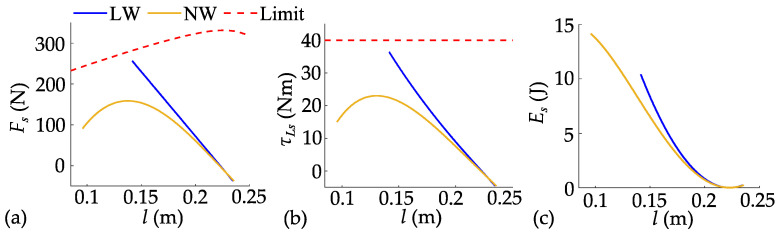
Comparison of planned results between two models: (**a**) spring force; (**b**) hip joint torque; (**c**) elastic potential energy.

Due to the lack of detailed literature describing the jumping methods of advanced robots like Ascento, as well as the unavailability of their dynamic parameters, it is not feasible to make a fair comparison with them. Given this situation, the widely used LW-SLIP model has become the most suitable comparison benchmark. This section compares the advantages of the NW-SLIP model in enhancing the robot’s jump height through jumps without leg retraction. Only the trajectory of the thrust stage is planned by solving the optimal control problem shown in (8), while the legs remain stationary afterward. Taking *h_s_* = 0.02 m as an example, the planned spring forces, hip torques, and elastic potential energies of the two models are shown in Figure 9.

**Figure 10:**
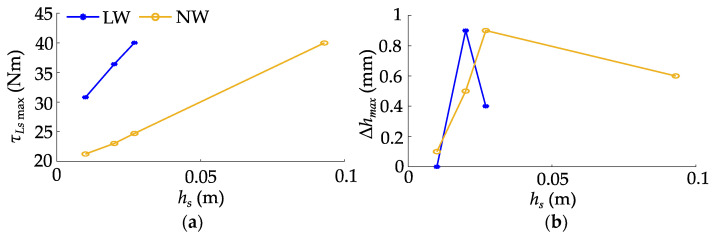
Comparison of simulation results based on two models: (**a**) maximum planned hip joint torque; (**b**) jump height error.

To achieve the same jump height, both models must generate an equivalent amount of kinetic energy. However, the NW-SLIP model enables flexible adjustment of the spring length at maximum force, better aligning the force curve with the joint output limit. The planned trajectory benefits from a longer acceleration distance, which significantly reduces the maximum torque demand on the joints, thereby facilitating trajectory tracking. From an energy perspective, the NW-SLIP model allows for greater storage of elastic potential energy with a lower maximum hip joint torque. This adaptability is crucial for the NW-SLIP model to fully harness the robot’s jumping potential, resulting in a substantial increase in achievable jump height. Then, *h_s_* is set to 0.01 m, 0.02 m, and 0.027 m, respectively, to further compare the two models, as shown in Figure 10. The maximum joint torque increases as target jump height increases. Without leg retraction, the maximum jump heights based on the two models are 0.027 m and 0.093 m, respectively.

NW-SLIP model demonstrated the full capacity of the hip joint and resulted in a jump height more than three times that of the LW-SLIP.

### 5.2. Simulation of Jumping Under Complete Trajectory

**Figure 11:**
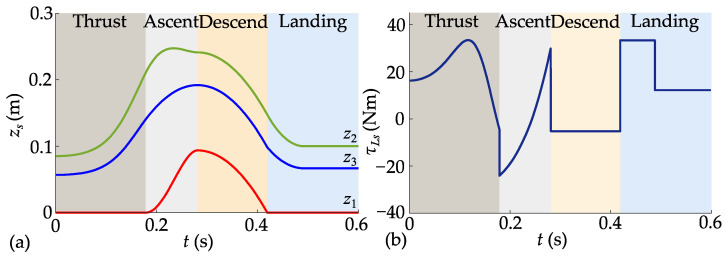
Planned trajectory for 0.093m jump height: (**a**) planned trajectory of centroid; (**b**) planned hip joint torque.

Taking *h_s_* = 0.093 m as an example, the planned complete trajectory is shown in Figure 11.

**Figure 12:**
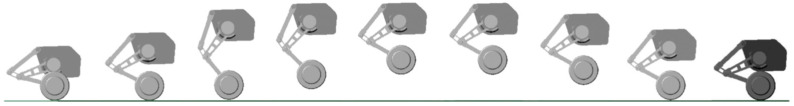
Simulated jumping effect with the complete trajectory at a target height of 0.93 m.

The positions and velocities of the upper and lower masses in the vertical direction remain consistent across stage transitions to ensure trajectory continuity. Sudden changes in joint torque at stage switching enhance the flexibility of trajectory planning, thereby contributing to an increase in jump height. The jumping performance achieved by tracking the complete trajectory is illustrated in Figure 12.

**Figure 13:**
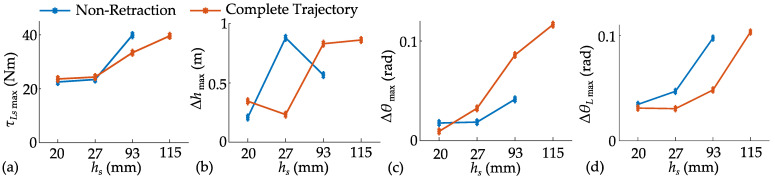
Jumping simulation under different target heights: (**a**) maximum planned joint torque; (**b**) jump height error; (**c**) maximum pitch angle deviation; (**d**) maximum joint angle deviation.

The jumping effects at different target heights are tested and further compared with the results without leg retraction, as shown in Figure 13. The jump height error can consistently be kept within 1 mm. Leg retraction reduces the torque required by the hip joint and increases the maximum jump height from 0.093 m to 0.115 m. Notably, the jump trajectory without leg retraction requires the upper and lower masses to instantaneously match velocities at *t*_1_, which introduces a risk of the legs exceeding their extension limits during actual jumps. When planning the complete trajectory, leg extension during the thrust stage is constrained by *c_l_*. This prevents the legs from overextending but reduces the acceleration distance for the upper mass, resulting in an insignificant increase in maximum jump height.

### 5.3. Simulation of Jumping onto a Step

**Figure 14:**
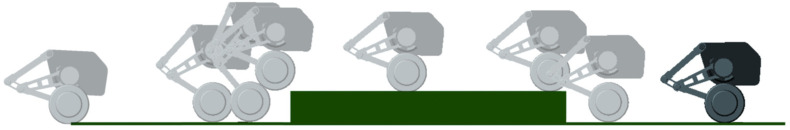
Simulation result of jumping onto a step.

Taking a 0.1 m height step as an example, the jump trajectory is planned based on the NW-SLIP model, and the required takeoff velocity and position are calculated accordingly. Notably, the LW-SLIP model can only generate a maximum jump height of 0.058 m, which is insufficient to clear the step successfully. The robot starts to jump when it reaches the starting position under the preset speed. The proposed jump control method is applied only for ascending the step, while step descent is handled by conventional walking, as shown in Figure 14.

**Figure 15:**
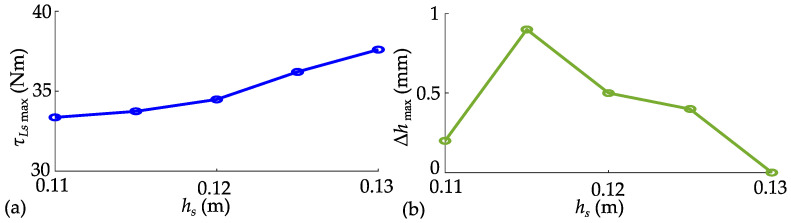
Simulation results of jumping onto a step under different jump heights: (**a**) maximum planned hip joint torque; (**b**) jump height error.

The target jump height is set to 0.1 m, enabling the robot to successfully jump onto the step and maintain stability. The ability to jump onto a step is further tested at different target jump heights, as shown in Figure 15.

It is demonstrated that the robot can successfully jump onto the step at different target heights. As the target height increases, the airtime lengthens, and the required speed decreases, which facilitates the completion of the jump. The jump height error can still be controlled within 1 mm, indicating that the proposed jump control method effectively ensures the accuracy of jump height during movement.

## 6. Jump Experiments on the Designed WBR

In this section, the simulation tests are repeated on the designed WBR system to prove the feasibility of the NW-SLIP model and jump control method.

### 6.1. Experiments of Jumping Without Leg Retraction

**Figure 16:**
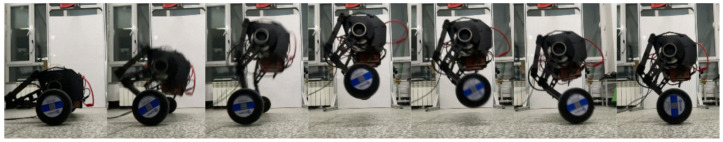
Diagram of jumping effect without leg retraction.

The trajectory is planned in the same way as in the simulation tests, and the jumping effect without leg retraction is shown in Figure 16.

**Figure 17:**
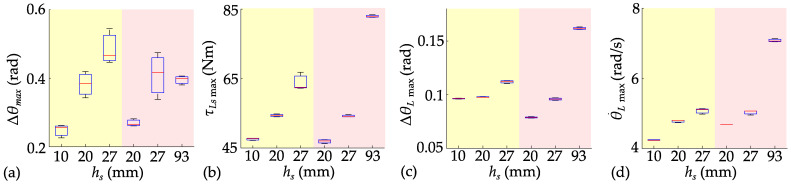
Jumping results without leg retraction: (**a**) maximum pitch angle deviation; (**b**) maximum planned hip torque; (**c**) maximum hip angle deviation; (**d**) maximum hip angular velocity.

The target jump heights are set to 0.01 m, 0.02 m, 0.027 m, and 0.093 m for real-time comparison of the LW-SLIP and NW-SLIP models. Repeat the jump three times for each height, and collect the information on fuselage pitch angle, joint torque, and jump height, as shown in Figure 17.

The sensor noise during the experiment caused the robot to remain in a dynamic adjustment state, resulting in unavoidable pitch angle deviations at takeoff. This led to increased fluctuations in the robot’s pitch angle throughout the jump. Furthermore, noise in the joint sensor impacted the trajectory tracking, which in turn affected the consistency of the jump. As a result, the experimental outcomes did not fully match the ideal performance observed in the simulations, although they exhibited a similar trend. Experimental results validate that the NW-SLIP model fully utilizes joint output to achieve a 3.4-fold increase in jump height. As jump height increases, the maximum hip joint angle deviation, angular velocity, and joint torque all rise correspondingly. The increased airtime similarly leads to a greater maximum pitch angle deviation. However, at the same jump height, the NW-SLIP model enables a longer acceleration distance and requires less joint torque, resulting in consistently lower deviations across all metrics and facilitating more accurate trajectory tracking.

### 6.2. Experiments of Jumping Under Complete Trajectory

**Figure 18:**
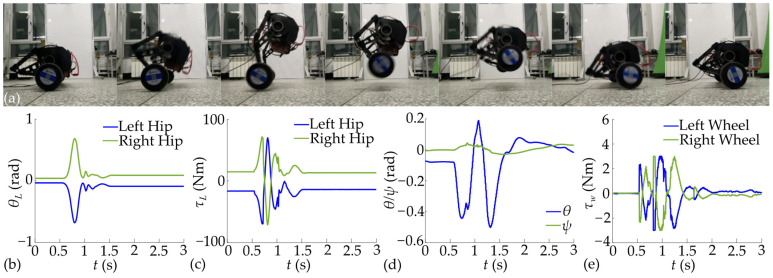
Experimental results of jumping under complete trajectory: (**a**) jumping diagram; (**b**) hip joint angle; (**c**) hip joint torque; (**d**) fuselage attitude angle; (**e**) wheel torque.

Taking *h_s_* = 0.093 m as an example again, the jump under complete trajectory is tested and shown in Figure 18.

**Figure 19:**
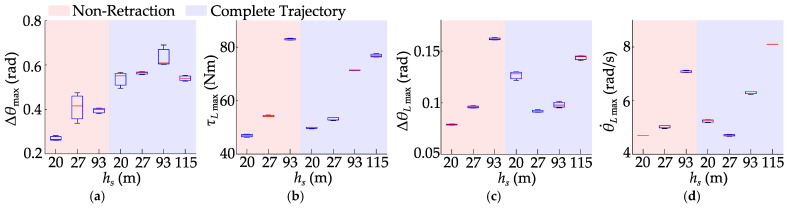
Experimental results of complete jumps under different target jump heights: (**a**) maximum pitch angle deviation; (**b**) maximum planned hip torque; (**c**) maximum hip angle deviation; (**d**) maximum hip angular velocity.

The designed WBR started to jump at 0.5 s, reached the preset height, and restored self-balance after landing. The maximum variation in the fuselage pitch angle is 0.63 rad during the flight and 0.69 rad after landing. The maximum torque of the hip joint is 71.49 Nm, and the maximum torque of the wheel is 3 Nm. A comparison between the jumping effect with and without leg retraction is conducted at different target jump heights, as shown in Figure 19.

Due to the inertia of the wheels, leg retraction increases the variation amplitude of the fuselage pitch angle during the jump, but it can still be controlled within 0.8 rad. As mentioned above, in the trajectory without leg retraction, the velocities of the upper and lower masses are assumed to be synchronized at the instant of *t*_1_. This makes the trajectory difficult to track, leading to an increase in the actual torque of the hip joint and maximum hip angle deviation at the same height. Maximum hip angular velocity generally increases with the jump height, but the maximum angular velocity at a jump height of 0.027 m for the complete trajectory is smaller than that of 0.02 m jump height. This is because the computational accuracy results in a greater proportion of the target ascent height in the trajectory for the 0.027 m jump height, which is also reflected in the maximum hip angle deviation.

### 6.3. Experimental Jumping onto the Steps

**Figure 20:**
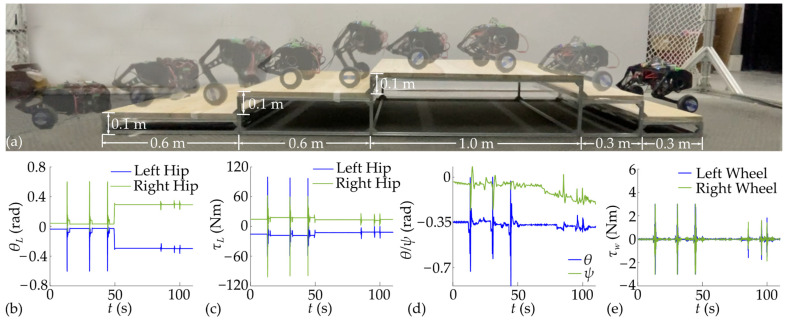
Experimental results of jumping onto the steps: (**a**) jumping diagram; (**b**) hip joint angle; (**c**) hip joint torque; (**d**) fuselage attitude angle; (**e**) wheel torque.

In this section, the designed WBR was controlled to jump onto and descend from the three steps, as shown in Figure 20. Each step had a height of 0.1 m, the width of the step to be jumped onto was 0.6 m, and the width of the step to be jumped from was 0.3 m. Setting the target jump height to 0.12 m, the robot successfully jumped onto and down three steps.

**Figure 21:**
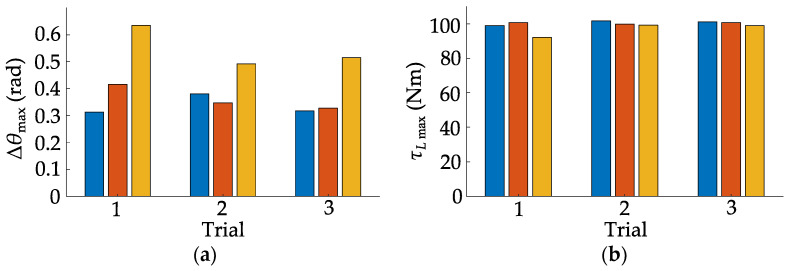
Summary of jumping onto the steps experiment results: (**a**) maximum pitch angle deviation; (**b**) maximum hip joint torque.

During multiple jumps, the maximum variation amplitude of the fuselage pitch angle ranges from 0.35 rad to 0.49 rad, and the maximum hip joint torque ranges from 99.24 Nm to 101.73 Nm. However, given the considerable height of the step for the robot, greater accuracy in speed, position, and yaw angle at the start of the jump is required. Coupled with the drift in yaw angle, the robot needs to be adjusted before each jump. Furthermore, the relatively small descent of the robot’s overall centroid during the descending stage reduces the deceleration distance required in the landing stage. This, in turn, allows for an increased retractable range of the legs during the ascent stage. Consequently, while the maximum height achieved in a stationary jump is only 0.115 m, the robot is able to jump onto the step with a preset height of 0.12 m. The experiment of jumping onto the steps will be repeated multiple times to thoroughly validate the jump control algorithm, as shown in Figure 21.

In the repeated experiments, the fuselage pitch angle fluctuated between 0.31 rad and 0.64 rad, and the maximum hip joint torque ranged from 92.18 Nm to 101.73 Nm. The fuselage pitch angle is influenced by movement speed, resulting in relatively larger fluctuations. However, the robot successfully and repeatedly jumps onto the steps multiple times, demonstrating the stability and consistency of the proposed jump control method.

## 7. Discussion

While the proposed trajectory planning and control method significantly enhances the jump height of WBRs, there are several limitations that require further research and optimization. Firstly, this study utilizes the cubic term NW-SLIP model for jump trajectory planning, with the aim of minimizing the maximum joint torque. In future work, higher-order stiffness coefficients could be introduced to further enhance the flexibility of the design. Additionally, energy constraints could be incorporated into the cost function to reduce energy consumption during the jump, thereby improving the robot’s endurance. Secondly, sensor noise, latency, ground compliance, and mass errors all have significant impacts on the precision and stability of jumping. Sensor noise introduces measurement inaccuracies that can compromise state estimation and interfere with the control system’s decision-making. Sensor latency further exacerbates the issue by introducing delays in feedback, which negatively impacts the system’s responsiveness and dynamic stability. These factors impose fundamental constraints on controller gain tuning. Excessively high gains may amplify the effects of noise and delay. Conversely, overly conservative gain settings can degrade the robot’s ability to maintain balance and accurately track the desired trajectory. As a result, both the accuracy and consistency of the jump performance may be adversely affected. Ground compliance is the most complex factor, as the reaction forces from different surfaces influence the robot’s takeoff power and landing stability. Soft or uneven ground can lead to instability. Mass errors affect the robot’s dynamic properties, which in turn influence both the jump height and the stability of posture during the jump. Although the trajectory tracking has been optimized by incorporating feedforward torque through a leg statics model and validated the consistency and stability of the algorithm through repeated experiments on two different surfaces (concrete and wood), these issues still require further investigation. Developing a more robust jump control algorithm that can adapt to these influences is crucial for enhancing the robot’s adaptability to diverse environments. Thirdly, from a hardware perspective, further structural optimization, weight reduction design, and the integration of torsional spring energy storage devices could further enhance the robot’s jumping performance. Additionally, analyzing battery consumption during consecutive jumps is also significant for improving the robot’s actual jumping performance. Moreover, from a functional perspective, the ability to adjust the robot’s posture in mid-air could enable diagonal jumps, further improving the robot’s actual jumping performance. Finally, research on the NW-SLIP model is still in its early stages. The flexibility and high energy storage advantages exhibited by the nonlinear model can further enhance the robot’s jumping performance and may also be extended to the control of other complex functions or jumping control of multi-legged robots.

## 8. Conclusions

This paper focuses on enhancing the jumping ability of WBRs to improve their environmental adaptability. The newly proposed NW-SLIP model, with simulated human-like force output strategies, achieves a jump height 3.4 times greater than that of the LW-SLIP model. Cost functions are established based on the virtual model, and the trajectories for each stage are iteratively optimized using QP and the bisection method. Minimizing the required joint torque while setting strict constraints aims to enhance the robot’s ability to perform jumps in real-world environments. Consequently, the maximum jump height is increased by 21.5%, while the maximum joint torque for the same height is reduced by 14%. In trajectory tracking control, alongside the kinematics model, a leg statics model is employed to compute the feedforward joint torque. This approach not only ensures precise trajectory tracking but also mitigates the uncertainties and complex calculations inherent in the dynamic model, demonstrating excellent potential for real-time applications. The proposed trajectory planning and control method performs well in jump consistency and stability, as evidenced in both simulations and experiments. Future work will further delve into the dynamic characteristics of the NW-SLIP model to enhance jumping performance and extend its application to other complex tasks. To further enhance the robot’s jumping performance in real-world environments, future work will investigate the effects of disturbances such as sensor noise, latency, and ground compliance. Robust control algorithms will be developed accordingly to mitigate these effects.

## Figures and Tables

**Table 1:** Definition of jump parameters.

Variable	Definition
*x*_1_, *z*_1_	X and z-coordinate of lower mass (m)
*x*_2_, *z*_2_	X and z-coordinate of upper mass (m)
*x*_3_, *z*_3_	X and z-coordinate of overall mass (m)
*t*_0_-*t*_4_	Switching moment of each stage (s)
*l_s_*, *l*	Target and actual length of the virtual spring (m)
*l*_0*t*_, *l*_0*l*_	Spring original length of thrust and landing stage (m)
*F_s_*	Planned spring force (N)
*F_t_*	Trajectory tracking force (N)
*F*	Total spring force (N)
*θ_s_*, *θ*	Target and actual pitch angle of fuselage (rad)
*τ_w_*	Wheel output torque (Nm)
*h_s_*	Target jump height (m)
*h_t_*	Target thrust height (m)
*h_a_*	Target ascent height (m)
*a_t_*	Cubic stiffness coefficient of thrust stage (N·m^−3^)
*b_t_*	Linear stiffness coefficient of thrust stage (N·m^−1^)
*a_l_*	Cubic stiffness coefficient of landing stage (N·m^−3^)
*b_l_*	Linear stiffness coefficient of landing stage (N·m^−1^)

**Table 2:** Summary of variables involved in trajectory planning.

Variable	Definition
*E_s_*(*a*, *b*, *l*, *l*_0_, *t*)	Total energy of the NW-SLIP model
∆*E_s_*(*a*, *b*, *l*, *l*_0_, *t*)	Total energy error function
*G*(*F_s_*, *l*_0_)	Support force coefficient
*Q*, *N*	State space of *q* and *F_s_*
*F*_max_	Maximum spring force corresponding to spring length
*c_l_*	Spring extension constraint coefficient
*L*(*c_i_*, *t*)	State variable error
*H*_1_(*a*, *b*, *l*, *l*_0_)	Constraints of the NW-SLIP model
*H*_2_(*z*_1_, *c_i_*, *t*)	Constraints of the cubic interpolation

## Data Availability

The data for all the figures in this paper are provided in the Supplementary Materials.

## Supplementary Materials

The supplementary materials can be downloaded at: https://www.mdpi.com/article/10.3390/biomimetics10040246/s1.
